# Short-term effects of fine particulate matter and ozone on the cardiac conduction system in patients undergoing cardiac catheterization

**DOI:** 10.1186/s12989-018-0275-z

**Published:** 2018-10-11

**Authors:** Siqi Zhang, Susanne Breitner, Wayne E Cascio, Robert B Devlin, Lucas M Neas, David Diaz-Sanchez, William E Kraus, Joel Schwartz, Elizabeth R Hauser, Annette Peters, Alexandra Schneider

**Affiliations:** 1 Institute of Epidemiology, Helmholtz Zentrum München, Ingolstädter Landstr. 1, P.O. Box 11 29, D-85764 Neuherberg, Germany; 2 National Health and Environmental Effects Research Laboratory, US Environmental Protection Agency, Research Triangle Park, Durham, NC USA; 3 Duke Molecular Physiology Institute, School of Medicine, Duke University, Durham, NC USA; 4 Department of Environmental Health, Harvard T.H. Chan School of Public Health, Boston, MA USA

**Keywords:** Air pollution, Electrocardiogram, PR interval, QT interval, QRS interval

## Abstract

**Background:**

Air pollution-induced changes in cardiac electrophysiological properties could be a pathway linking air pollution and cardiovascular events. The evidence of air pollution effects on the cardiac conduction system is incomplete yet. We investigated short-term effects of particulate matter ≤ 2.5 μm in aerodynamic diameter (PM_2.5_) and ozone (O_3_) on cardiac electrical impulse propagation and repolarization as recorded in surface electrocardiograms (ECG).

**Methods:**

We analyzed repeated 12-lead ECG measurements performed on 5,332 patients between 2001 and 2012. The participants came from the Duke CATHGEN Study who underwent cardiac catheterization and resided in North Carolina, United States (NC, U.S.). Daily concentrations of PM_2.5_ and O_3_ at each participant’s home address were predicted with a hybrid air quality exposure model. We used generalized additive mixed models to investigate the associations of PM_2.5_ and O_3_ with the PR interval, QRS interval, heart rate-corrected QT interval (QTc), and heart rate (HR). The temporal lag structures of the associations were examined using distributed-lag models.

**Results:**

Elevated PM_2.5_ and O_3_ were associated with four-day lagged lengthening of the PR and QRS intervals, and with one-day lagged increases in HR. We observed immediate effects on the lengthening of the QTc interval for both PM_2.5_ and O_3_, as well as delayed effects for PM_2.5_ (lagged by 3 – 4 days). The associations of PM_2.5_ and O_3_ with the PR interval and the association of O_3_ with the QRS interval persisted until up to seven days after exposure.

**Conclusions:**

In patients undergoing cardiac catheterization, short-term exposure to air pollution was associated with increased HR and delays in atrioventricular conduction, ventricular depolarization and repolarization.

**Electronic supplementary material:**

The online version of this article (10.1186/s12989-018-0275-z) contains supplementary material, which is available to authorized users.

## Background

The associations between ambient air pollution and cardiovascular morbidity and mortality are well established [1–3]. One potential pathway of the linkage might be through the air pollution-induced changes in cardiac electrophysiological properties. The cardiac conduction system initiates and conducts electrical impulses as recorded in the electrocardiogram (ECG). Cardiac conduction abnormalities, such as first-degree atrioventricular block (first-degree AVB) or prolonged ventricular repolarization, are associated with increased incidence and prevalence of atrial fibrillation (AF), total mortality, and sudden cardiac death [4, 5].

The acute effects of air pollution on the cardiac conduction system could be mediated by physiological mechanisms including autonomic imbalance and systemic inflammation, which trigger both immediate and delayed responses over a period ranging from hours to days [6, 7]. Epidemiological studies have reported associations of a lengthening of the heart rate-corrected QT interval (QTc), a measure of ventricular repolarization, with particulate matter in the elderly and patients having diabetes or preexisting ischemic heart disease [6, 8–11]. However, such associations were not observed in a panel study of cardiac rehabilitation patients [7]. In addition to the inconsistent results of particulate matter, evidence of ambient ozone (O_3_) effects on the QTc interval is still limited [12, 13], and the impacts of air pollution on atrioventricular conduction and ventricular depolarization have not been fully investigated [9, 14].

Hypothesizing that air pollution exposure would be associated with cardiac conduction delay, we performed this study to investigate the short-term effects of PM_2.5_ and O_3_ on the electrocardiographic intervals reflecting impulse propagation and repolarization in high-risk patients from a cardiovascular cohort.

## Methods

### Study population

The data used in this study were obtained from the Catheterization Genetics (CATHGEN) Study, a cohort of 9,334 patients who underwent cardiac catheterization at Duke University Medical Center from 2001 through 2010. More details of the CATHGEN Study can be found elsewhere [15].

Our analyses were restricted to 6,209 individuals who had ECG measurements and resided in North Carolina, United States (NC, U.S.) at catheterization. From the CATHGEN database, we collected information on participant demographic characteristics (age, sex, and race), body mass index (BMI), smoking status, and the history of myocardial infarction (MI). The Coronary Artery Disease Prognostic index (CAD index) was assessed during the catheterization procedure. The CAD index is an indicator of the severity of coronary artery disease (CAD) based upon cardiovascular outcomes. A CAD index > 23 represents at least one ≥ 75% occlusion in one major epicardial coronary artery [16]. Data on area-level educational attainment and urban/rural status were obtained from the 2000 U.S. Census based on each patient’s home address at catheterization. Area-level educational attainment refers to the percentage of adults (≥ 25 years old) in the block group with less than a high school education; it was categorized into low (≥ 25%) and high (< 25%) levels.

### ECG measurement

During the study period (2001–2012), 71,194 12-lead ECGs were performed at the time of catheterization and in follow-up examinations, and analyzed automatically using the Philips TraceMaster ECG system (Andover, MA). ECG parameters of interest were the PR interval (ms), QRS interval (ms), QT interval (ms), and heart rate (HR, beats/min). The PR interval is measured from the beginning of the P wave to the beginning of the QRS complex, reflecting the electrical impulse conduction from the sinus node through the atrioventricular node and His-Purkinje system. The QRS interval is the time from the onset of the Q wave to the end of the S wave, which represents ventricular depolarization. The QT interval is defined as the duration from the beginning of the Q wave to the end of the T wave. The QT interval is dependent on HR; after HR-correction, the QTc interval is a measure of ventricular repolarization. The QT correction for HR was performed using the Bazett formula in our main analyses [17].

We first excluded 13,632 ECGs with the diagnosis of atrial fibrillation, atrial flutter, multifocal atrial tachycardia, or paced rhythms. For participants with multiple ECGs on the same day or ECGs on consecutive days, we only included the first of the day and the first on consecutive days to reduce the potential impact of intervening medical treatment. To reduce bias caused by artifacts, we excluded ECGs with non-physiological parameter values in the following ranges: (1) PR interval < 100 ms or > 400 ms, (2) QRS interval < 50 ms or > 170 ms, (3) QTc < 350 ms or > 600 ms, (4) HR < 20 beats per minute (bpm) or > 180 bpm. We further excluded participants with bundle branch block (BBB, QRS interval > 120 ms), leaving 28,741 eligible ECGs on 5,376 participants.

### Exposure assessment

Daily concentrations of PM_2.5_ (daily average in μg/m^3^) and O_3_ (daily 8-h maximum in ppb) for NC were predicted at a 1 km × 1 km spatial grid resolution from 2000 to 2012. Predictions were made using a neural network-based hybrid model, incorporating input variables such as chemical transport model outputs, satellite-based aerosol optical depth data, absorbing aerosol index, land-use terms, and meteorological variables. The ten-fold cross-validation indicated good model performances with coefficients of determination of 0.86 and 0.68 for PM_2.5_ and O_3_, respectively. Detailed descriptions and predictive performance of the model were reported elsewhere [18, 19].

Daily air temperature in NC was also predicted at a 1 km × 1 km grid resolution for the study period. The modeling process involved satellite-derived daily surface temperature, daily air temperature from NC weather stations, normalized difference vegetation index, and predictors of air temperature (percent of urban areas, elevation, and distance to water body). A three-stage modeling approach was used, allowing the prediction in grid cells without weather monitors or grid cells/days without data on satellite surface temperature [20].

The latitude and longitude of each participant’s residential address were geocoded by the Children’s Environmental Health Initiative in the Duke Nicholas School of the Environment (https://cehi.rice.edu/). For individuals who moved during the study period, we used the address most closely linked with the date on which the ECG was performed. The geocoded addresses were matched with air pollution and temperature data based on the spatial location and date. Daily air pollutant concentrations and air temperature on the same day and 1–14 days prior to the ECG measurement were assigned to each ECG.

### Statistical analysis

Short-term effects of PM_2.5_ and O_3_ on ECG parameters were investigated using generalized additive mixed models with random intercepts for patients. The ECG parameters were log-transformed in our regression models to increase the conformity to a normal distribution of residuals. To control for systematic variation over time, we included a penalized spline for long-term time trend with four degrees of freedom per year, and two categorical variables for season (spring: March–May; summer: June–August; autumn: September–November; winter: December–February) and day of the week. Air temperature was adjusted for by modeling low and high temperatures separately [21]. For days with average temperature on the previous four days (lag1–4) lower than the median annual temperature, we introduced a natural spline with two degrees of freedom for lag1–4 temperature. Similarly, for days with average temperature on the current and previous day (lag0–1) higher than the median annual temperature, we introduced a natural spline for lag0–1 temperature with three degrees of freedom. Besides, we controlled for individual characteristics at each measurement time point, including age (continuous), sex (male or female), race (European-Americans, African-Americans, or others), area-level educational attainment (low or high), BMI (continuous), smoking status (never smoker, or current/former smoker), and living area (rural or urban). The adjusted confounders were identical across models for the various air pollutants and ECG parameters. We investigated the effects of air pollution on the concurrent day (lag0), for single-day lags from one to four days (lag0–lag4), and for a multi-day lag of five days (lag04).

For pollutant-outcome pairs showing significant delayed associations four days after exposure, we examined lagged effects up to 14 days using distributed-lag models [22]. We therefore built a cross-basis matrix with a third degree polynomial function of lags, which was then incorporated into the generalized additive mixed model adjusted for the same confounders as in the main model.

To explore effect modification and identify the subgroups that might be more susceptible to the effects of PM_2.5_ and O_3_, we incorporated interaction terms between air pollution and individual characteristics in the model. The examined potential modifiers included sex, age (< 60 years vs. ≥ 60 years), area-level educational attainment, obesity (BMI < 30 kg/m^2^ vs. ≥ 30 kg/m^2^), smoking status, urban/rural status, CAD index (CAD index ≤ 23 vs. > 23), and history of MI.

In sensitivity analyses, we excluded ECGs with single-day (lag0–lag4) exposure levels of PM_2.5_ above 35 μg/m^3^ or O_3_ above 70 ppb to examine the effects of air pollution below the current U.S. National Ambient Air Quality Standards (NAAQS) [23]. As the electrophysiological parameters are potentially dependent on the HR, we further adjusted for the HR in models for the PR interval, QRS interval, and the raw QT interval without HR-correction. In addition, we used Fridericia formula in QT correction [24], and investigated air pollution effects on corrected JT interval (JTc), which was defined by subtracting the QRS from QTc. The JTc interval is also an indicator to measure the duration of ventricular repolarization and is reported to reduce the impact of wide QRS complex on the QTc interval [25]. To examine the influence of BBB on associations between air pollution and ECG parameters, we performed analyses using 33,117 eligible ECG measurements on 5,819 participants regardless of the presence of BBB. We tested the robustness of the results by building two-pollutant models with PM_2.5_ and O_3_ of the same lag, restricting the analyses to participants with two or more ECG measurements, changing the degree of freedom for the trend spline, excluding season as a categorical variable, and applying generalized additive mixed models with linear spatial correlation structure given that the dependency between repeated ECG measurements might decrease with increasing time interval. Furthermore, we added long-term air pollution exposure (365-day moving average of air pollution of 0–364 days prior to each ECG measurement) to our models and replaced the daily mean concentration with the deviation between daily mean and long-term average. In this way, we sought to investigate the acute effect of temporal variation of pollutants with the control for spatial variation. The linearity of the exposure-response relationships was examined by including a spline for air pollution variables in models.

The effect estimates are reported as percent changes of the geometric mean (GM) of outcomes and 95% confidence intervals (95% CI) corresponding to an interquartile range (IQR) increase in PM_2.5_ and O_3_. We performed the analyses with the software R (version 3.5.1), using the ‘gamm4’, ‘mgcv’, and ‘dlnm’ packages. The significance level was set at 0.05.

## Results

### Participant characteristics and exposure concentrations

After further exclusion of 44 patients without complete data on ECG parameters of interest, air pollution concentrations, or main covariates, we analyzed a final sample of 28,578 ECGs on 5,332 participants (See Additional file 1: Figure S1). Among them, 4,009 participants had two or more eligible ECG recordings during the study period. The mean age and BMI at enrollment were 59.8 years and 30.1 kg/m^2^, respectively (Table 1). 60.7% of the participants were male, over half were never smokers, and the majority were European-American (72.3%). More individuals lived in rural areas and areas with a high level of educational attainment. Compared to excluded individuals, the participants included in our main analyses tended to be younger and more likely to live in urban areas, have a higher proportion of African-Americans and a higher level of educational attainment (See Additional file 1: Table S1).

**Table 1:** Descriptive statistics of the study population at baseline (*n*=5332)

	Mean ± SD / N (%)
Age (years)	59.8 ± 11.7
BMI (kg/m^2^)	30.1 ± 7.2
Sex (male)	3237 (60.7)
Race	
European-Americans	3854 (72.3)
African-Americans	1188 (22.3)
Others	290 (5.4)
Smoking (never smoker)	2753 (51.6)
Education (high)	3231 (60.6)
Area (rural)	2953 (55.4)
CAD-index > 23 (yes)^a^	2418 (50.4)
History of MI (yes)	1449 (27.2)

*SD* standard deviation, *BMI* body mass index, *CAD* coronary artery disease, *MI* myocardial infarction

^a^Data on CAD-index were available for 4801 participants

Table 2 shows the descriptive statistics of ECG parameters in all ECG recordings. The correlations between ECG parameters were weak or negligible. During the study period, the average concentrations of PM_2.5_ and O_3_ in geocoded areas with participants were 11.2 μg/m^3^ and 40.5 ppb, respectively (Table 3). Most daily PM_2.5_ and O_3_ levels (99.9% for PM_2.5_ and 98.7% for O_3_) were below the current NAAQS (daily average concentration of 35 μg/m^3^ for PM_2.5_ and daily maximum 8-hour concentration of 70 ppb for O_3_). PM_2.5_ and O_3_ were moderately correlated with a Spearman correlation coefficient of 0.49.

**Table 2:** Descriptive statistics and Spearman correlation coefficients of ECG parameters (n=28578)

	Mean ± SD		Min	25%	Median	75%	Max	Correlation coefficients		
	Geometric	Arithmetic						PR	QRS	QTc
PR (ms)	170 ± 1	173 ± 31	100^a^	152	168	188	400^a^	--		
QRS (ms)	91 ± 1	91 ± 12	53	83	90	99	120^a^	0.18	--	
QTc (ms)	434 ± 1	435 ± 33	350^a^	412	433	456	587	-0.02	0.24	--
HR (bpm)	73 ± 1	74 ± 17	31	62	72	85	160	-0.28	-0.09	0.32

*SD* standard deviation, *Min* minimum, *25%* the 25th percentile, *75%* the 75th percentile, *Max* maximum, *QTc* heart rate-corrected QT interval, *HR* heart rate, *bpm* beats per minute

^a^The minimum and maximum values were set by the exclusion criteria

**Table 3:** Descriptive statistics of air pollution and temperature in geocoded areas with participants during the study period

	Mean ± SD	Min	25%	Median	75%	Max	IQR
PM_2.5_ (μg/m^3^)	11.2 ± 5.5	0.9	7.3	10.1	14.2	54.5	7.0
O_3_ (ppb)	40.5 ± 12.8	8.6	30.4	39.5	49.8	97.6	19.4
Air temperature (°C)	16.8 ± 8.1	-4.7	10.2	17.8	24.0	31.0	13.8

*SD* standard deviation, *Min* minimum, *25%* the 25th percentile, *75%* the 75th percentile, *Max* maximum, *IQR* interquartile range, *PM*_*2.5*_ particulate matter ≤ 2.5 μm in aerodynamic diameter, *O*_*3*_ ozone, *ppb* parts per billion

### Air pollution and ECG parameters

Increments in PM_2.5_ and O_3_ were significantly associated with the lengthening of the PR interval lagged three or four days, and with the concurrent as well as lagged lengthening of the QTc interval (Table 4). Positive associations with the QRS interval were significant for O_3_ at lag4 and marginally significant for PM_2.5_ at lag1 and lag4. We also observed significant increases in the HR associated with elevated PM_2.5_ and a marginally significant increase for O_3_, with the strongest single-day effects at lag1.

**Table 4:** Percent change (95% CI) of the geometric mean of ECG parameters per interquartile range increase in pollutants

ECG parameter	Lag (day)	PM_2.5_	O_3_
		% Change (95% CI)	% Change (95% CI)
PR	0	-0.07 (-0.23, 0.08)	-0.01 (-0.24, 0.23)
	1	-0.07 (-0.23, 0.09)	0.03 (-0.21, 0.27)
	2	-0.07 (-0.23, 0.09)	-0.12 (-0.36, 0.12)
	3	0.17 (0.01, 0.33)^*^	0.02 (-0.22, 0.26)
	4	0.18 (0.03, 0.34)^*^	0.29 (0.05, 0.53)^*^
	04	0.07 (-0.18, 0.32)	0.09 (-0.26, 0.43)
QRS	0	0.11 (0.00, 0.22)	-0.04 (-0.21, 0.12)
	1	0.03 (-0.08, 0.14)	0.00 (-0.17, 0.16)
	2	0.01 (-0.10, 0.12)	-0.05 (-0.22, 0.11)
	3	0.04 (-0.07, 0.15)	0.04 (-0.13, 0.21)
	4	0.11 (0.00, 0.21)	0.21 (0.04, 0.37)^*^
	04	0.15 (-0.03, 0.32)	0.06 (-0.18, 0.30)
QTc	0	0.11 (0.02, 0.19)^*^	0.17 (0.04, 0.30)^**^
	1	0.05 (-0.04, 0.14)	0.18 (0.04, 0.31)^**^
	2	0.05 (-0.04, 0.14)	0.07 (-0.06, 0.21)
	3	0.11 (0.03, 0.20)^*^	0.02 (-0.11, 0.15)
	4	0.13 (0.05, 0.22)^**^	0.04 (-0.09, 0.17)
	04	0.23 (0.09, 0.36)^**^	0.20 (0.01, 0.39)^*^
HR	0	0.22 (-0.05, 0.49)	0.23 (-0.17, 0.63)
	1	0.47 (0.20, 0.75)^**^	0.40 (0.00, 0.81)
	2	0.28 (0.01, 0.56)^*^	0.28 (-0.13, 0.69)
	3	-0.11 (-0.38, 0.16)	-0.21 (-0.62, 0.20)
	4	0.04 (-0.23, 0.30)	-0.12 (-0.53, 0.29)
	04	0.44 (0.01, 0.86)^*^	0.24 (-0.34, 0.83)

*CI* confidence interval, *ECG* electrocardiogram, *PM*_*2.5*_ particulate matter ≤ 2.5 μm in aerodynamic diameter, *O*_*3*_ ozone; *QTc* heart rate-corrected QT interval, *HR* heart rate

^*^p-Value <0.05; ^**^p-Value <0.01

**Fig. 1:**
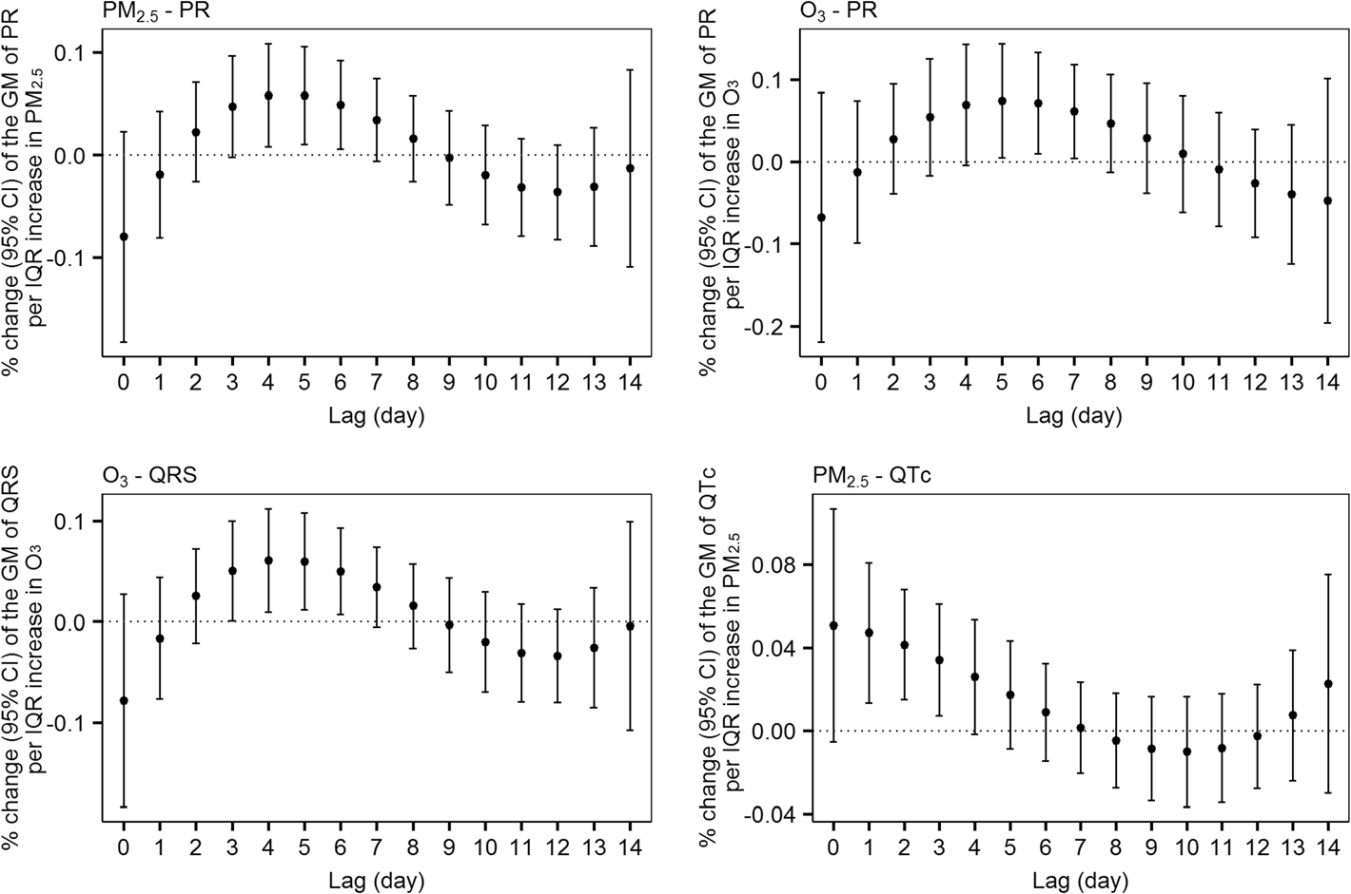
Percent change (95% CI) of the geometric mean of ECG parameters per interquartile range increase in PM_2.5_ and O_3_ in distributed-lag models. *CI* confidence interval, *ECG* electrocardiogram, *PM*_2.5_ particulate matter ≤ 2.5 μm in aerodynamic diameter, *O*_3_ ozone *QTc* heart rate-corrected QT interval, *GM* geometric mean, *IQR* interquartile range

We used polynomial distributed-lag models for PR, QRS, and QTc intervals as they showed delayed responses to air pollution. Estimates of the distributed-lag models indicated that the effects of PM_2.5_ and O_3_ on the PR interval and the effect of O_3_ on the QRS interval persisted until seven days after exposure. For the QTc interval, we did not find lagged effects of PM_2.5_ beyond four days (Fig. 1).

### Effect modification

We observed stronger effects of O_3_ on the QRS and QTc intervals in patients living in rural areas, and stronger air pollution effects on the QTc interval in patients with low educational attainment or obesity. We did not find significant or consistent effect modification by other examined potential modifiers (See Additional file 1: Figure S2).

### Sensitivity analyses

Analyses of exposure below the NAAQS showed slightly attenuated associations between air pollution and ECG parameters; the effects of air pollution on the PR interval, QTc interval, and HR remained significant (See Additional file 1: Table S2). Associations of air pollution with the PR, QRS, and QT intervals were not sensitive to the adjustment for HR (See Additional file 1: Figure S3). We observed reduced effects of air pollution on the QTc interval calculated using the Fridericia formula compared to using the Bazett formula at lag0–lag2 (See Additional file 1: Figure S4). However, the associations between air pollution and ventricular repolarization were generally consistent across different indicators. Including participants with BBB reduced the air pollution effects on the QRS and QTc intervals and did not significantly affect the effects on the PR interval and HR (See Additional file 1: Figure S5).

We did not observe substantial changes in effect estimates in two-pollutant models, except for the attenuated effect of PM_2.5_ on the PR interval at lag4 when adjusted for O_3_ and vice versa (See Additional file 1: Figure S6). The associations of PM_2.5_ and O_3_ with ECG parameters were robust to excluding participants with only one ECG measurements, changing the degree of freedom of trend spline, excluding season, controlling for long-term exposure to air pollution, or applying spatial correlation structure in mixed-effects models. The linear exposure-response relationships between air pollution and ECG parameters held true when air pollution variables were included in models as splines (results not shown).

## Discussion

In high-risk patients undergoing cardiac catheterization, we observed associations of increments in PM_2.5_ and O_3_ with the lengthening of the PR, QRS, and QTc intervals and increased HR. The effects of PM_2.5_ and O_3_ on the PR interval and the effect of O_3_ on the QRS interval persisted until up to one week in distributed lag models. These findings supported our hypothesis that short-term exposure to air pollution was associated with atrioventricular and intraventricular conduction delay.

An increased PR interval could relate to parasympathetic activation, sympathetic withdrawal, or the block of inward calcium current through membrane channels. A lengthening of the PR interval, even below the diagnostic threshold for first-degree AVB (PR interval > 200 ms), is associated with increased incidence of AF, pacemaker implantation, and all-cause mortality [5]. Few prior studies investigated the effect of air pollution on the PR interval. The Air Pollution and Cardiac Risk and its Time Course (APACR) Study found a 0.09% increase in the PR interval for each 10 μg/m^3^ increment in PM_2.5_ [14]. Since individuals with cardiovascular disease are potentially more sensitive to air pollution effects, the smaller effect estimate compared to our study (0.25%) could be due to the healthier participants in the APACR Study. The distinct lag times of associations (1.5–2 hours in the APACR Study and 3–4 days in our study) might also partly explain the difference. In addition to PM_2.5_, our study provided evidence for an association between O_3_ and the PR interval, which to our knowledge has not been reported previously.

The associations between air pollution and the QRS interval in our study indicated the effects of air pollution on ventricular depolarization among individuals without bundle branch block. An increase in the QRS interval is an independent predictor of cardiovascular mortality [26]. Yet, the evidence of air pollution effects on the QRS interval is still limited. Consistent with our results, a higher prevalence of prolonged QRS interval was associated with long-term residential PM_2.5_ exposure in the U.S. Multi-Ethnic Study of Atherosclerosis (MESA) Cohort. Besides, in a controlled exposure study among individuals with metabolic syndrome, the GSTM1 null participants showed an increased QRS interval after acute exposure to concentrated ambient ultrafine particles [27]. However, the QRS interval was not associated with PM_2.5_ in the APACR Study [9], and an immediate decrease of 5.8% (95%CI: -10.5, -1.0) in the QRS interval after exposure to O_3_ was observed in a crossover study among healthy volunteers [13]. The mechanism by which air pollution might lead to a change in QRS complex remains unclear and needs to be clarified by further epidemiological and experimental studies. Some theoretical explanations could be the impact of air pollution on the inward sodium current and the extracellular resistance.

Our findings of both concurrent and delayed effects of air pollution on the lengthening of the QTc interval are supported by previous studies [6, 8, 13]. The potential pathway of the immediate associations could be the direct impact of air pollution on the autonomic nervous system, and the delayed effects are possibly mediated by air pollution-induced inflammatory responses [6]. Ambient air pollutants trigger reactive oxygen species production, which in turn induces pulmonary and systemic inflammation. The concentrations of circulating inflammatory biomarkers, such as C-reactive protein (CRP), interleukin 6, and fibrinogen, are increased after acute air pollution exposure [6, 28]. Further, inflammation is a modulator of cardiomyocyte ion currents in the cardiac conduction system, through a pathway involving cytokine- and sympathetic-induced modulation [29]. Elevated levels of circulating inflammatory biomarkers have been proven to be associated with QTc prolongation [30–32].

The QTc interval calculated using the Bazett formula has been reported to be inferior to using the Fridericia formula in the prediction of mortality [33]. In our study, the associations between air pollution and the QTc interval calculated using the Fridericia formula were generally comparable to using the Bazett formula. Similar results were also found in the APACR Study [9]. In addition, since the QT interval encompasses the duration of ventricular depolarization as reflected by the QRS interval, the air pollution-induced lengthening of the QTc interval could be partly attributable to the effects on the QRS interval. When subtracting the QRS from the QTc, we still observed significant associations between air pollution and the JTc interval. The robust results provided strong evidence for the air pollution effects on ventricular repolarization.

The 1–2 days lagged associations between air pollution and HR suggested the effects of air pollution on the autonomic nervous system [8, 34–36]. These associations could be potentially affected by the use of medication. For example, stronger effects of air pollution on HR and heart rate variability are observed among individuals not taking beta-blockers or calcium-channel blockers [8, 34]. On the other hand, taking medication indicates the presence of underlying clinical conditions, which might increase individual’s susceptibility to air pollution. Therefore, other studies reported non-significant effect modification by medication or even stronger effects in individuals taking angiotensin-converting-enzyme inhibitor (ACE inhibitor) [37, 38]. The interaction between medication usage and clinical conditions potentially limits the interpretability of the non-significant effect modification by CAD in our study.

Although the effects of air pollution on the cardiac conduction system were relatively small in this study, it is still of public health significance because of its implications for the entire population. Using the World Health Organization air quality guideline for 24-hour mean of PM_2.5_ (25 μg/m^3^) as reference [39], exposure to the maximum PM_2.5_ in this study (54.5 μg/m^3^) would account for an increase of 2.4 ms in the QTc interval in exposed individuals. Moreover, cardiac conduction is affected by many other factors. For instance, preexisting medical conditions (left ventricular hypertrophy, ischemia, etc.) and certain medications can prolong cardiac repolarization [40]. Among patients with these conditions, further exposure to air pollution may add to the effects of other factors, and drive the QT interval across a critical threshold.

### Strengths and limitations

A major strength of this study is the large sample size of the study population and the vast number of ECG recordings for analyses, which to the best of our knowledge is the largest cohort for analyzing air pollution effects on ECG parameters. The repeated measures study design provided substantial statistical power and enabled control for unmeasured individual-level confounders. Besides, we investigated the associations of PM_2.5_ and O_3_ with ECG parameters that have rarely been examined previously, such as the PR and QRS intervals.

One limitation of the study is the heterogeneity of time intervals caused by unscheduled follow-up visits. In the analyses, we applied mixed-effects models, which can reduce the impact of the unbalanced data structure. Second, we used daily residential exposure assessment instead of personal exposure. This may have resulted in non-differential exposure misclassification and bias the results towards the null [41]. Third, due to the unavailability of data, we were not able to control for medication intake, and the smoking status was roughly divided into current/former smoker or never smoker, which may have led to inaccuracy in assessing effect modification by pre-existing morbidities and residual confounding. Finally, our study was performed in high-risk patients receiving cardiac catheterization; thus, the results may not be generalizable to the general population. However, it enabled us to assess the association in a population subgroup at greater risk of cardiovascular events and potentially more susceptible to the adverse effects of air pollution.

## Conclusions

In summary, short-term exposure to PM_2.5_ and O_3_ was associated with lengthening of the PR, QRS, and QTc intervals, and increasd heart rate in patients with cardiovascular disease. These findings provide evidence for the acute effects of air pollution on atrioventricular conduction and ventricular deporlarization and repolarization, which could potentially mediate the associations of air pollution with cardiac arrhythmias and cardiovascular mortality.

## Additional file

Additional file 1: **Table S1.** Comparison of individual characteristics between participants included and excludes in main analyses. **Table S2.** Percent change (95% CI) of the geometric mean of ECG parameters per interquartile range increase in PM_2.5_ and O_3_ below the NAAQS. **Figure S1.** Flow chart of the exclusion procedure. **Figure S2.** Effect modification (percent change with 95% CI) by participant characteristics on the associations of air pollution with ECG parameters. **Figure S3.** Percent change (95% CI) of the geometric mean of the PR, QRS, and raw QT intervals per interquartile range increase in PM_2.5_ and O_3_ in models with adjustment for HR. **Figure S4**. Comparison of the air pollution effects (percent change with 95% CI) on different ventricular repolarization indicators. **Figure S5.** Percent change (95% CI) of the geometric mean of ECG parameters per interquartile range increase in PM_2.5_ and O_3_ among participants with QRS ≤ 120 ms and participants with QRS in the full range (50 ms ≤ QRS ≤ 170 ms). **Figure S6.** Percent change (95% CI) of the geometric mean of ECG parameters per interquartile range increase in PM_2.5_ and O_3_ in sensitivity analyses. (DOC 884 kb)

## Abbreviations

AF: Atrial fibrillation
AVB: Atrioventricular block
BBB: Bundle branch block
BMI: Body mass index
BPM: Beats per minute
CAD: Coronary artery disease
CI: Confidence interval
CRP: C-reactive protein
ECG: Electrocardiogram
GM: Geometric mean
HR: Heart rate
IQR: Interquartile range
MI: Myocardial infarction
NAAQS: National Ambient Air Quality Standards
O_3_: Ozone
PM_2.5_: Particulate matter ≤ 2.5 μm in aerodynamic diameter
QTc: Heart rate-corrected QT interval
SD: Standard deviation
