# Supplementary material for: Short-term effects of fine particulate matter and ozone on the cardiac conduction system in patients undergoing cardiac catheterization
Source: Part Fibre Toxicol. 2018 Oct 11;15:38. doi: 10.1186/s12989-018-0275-z (PMC6180522; doi:10.1186/s12989-018-0275-z)
Supplement: Supplementary file 1 — Table S1. Comparison of individual characteristics between participants included and excludes in main analyses. Table S2. Percent change (95% CI) of the geometric mean of ECG parameters per interquartile range increase in PM2.5 and O3 below the NAAQS. Figure S1. Flow chart of the exclusion procedure. Figure S2. Effect modification (percent change with 95% CI) by participant characteristics on the associations of air pollution with ECG parameters. Figure S3. Percent change (95% CI) of the geometric mean of the PR, QRS, and raw QT intervals per interquartile range increase in PM2.5 and O3 in models with adjustment for HR. Figure S4. Comparison of the air pollution effects (percent change with 95% CI) on different ventricular repolarization indicators. Figure S5. Percent change (95% CI) of the geometric mean of ECG parameters per interquartile range increase in PM2.5 and O3 among participants with QRS ≤ 120 ms and participants with QRS in the full range (50 ms ≤ QRS ≤ 170 ms). Figure S6. Percent change (95% CI) of the geometric mean of ECG parameters per interquartile range increase in PM2.5 and O3 in sensitivity analyses. (DOC 884 kb) [file 12989_2018_275_MOESM1_ESM.doc]

**Table S1. Comparison of individual characteristics between participants included and excludes in main analyses.**

|  | Mean ± SD / N (%) | | *p*-value |
| --- | --- | --- | --- |
|  | Included participants  (n=5332) | Excluded participants  (n=1784) |
| Age (years) | 59.8 ± 11.7 | 62.8 ± 12.9 | <0.001 |
| BMI (kg/m^2^) | 30.1 ± 7.2 | 30.1 ± 7.5 | 0.90 |
| Sex (male) | 3237 (60.7) | 1131 (63.4) | 0.05 |
| Race |  |  | < 0.001 |
| European-Americans | 3854 (72.3) | 1369 (76.7) |  |
| African-Americans | 1188 (22.3) | 263 (14.7) |  |
| Others | 290 (5.4) | 152 (8.5) |  |
| Smoking (never smoker) | 2753 (51.6) | 901 (50.5) | 0.43 |
| Education (high) | 3231 (60.6) | 961 (53.9) | < 0.001 |
| Area (rural) | 2953 (55.4) | 1199 (67.2) | < 0.001 |
| CAD-index > 23 (yes)^a^ | 2418 (50.4) | 816 (49.5) | 0.56 |
| History of MI (yes) | 1449 (27.2) | 496 (27.8) | 0.63 |

SD: standard deviation; BMI: body mass index; CAD: coronary artery disease; MI: myocardial infarction.

### ^a^ Data on CAD-index were available for 5246 included participants and 1203 excluded participants.

### **Table S2.** Percent change (95% CI) of the geometric mean of ECG parameters per interquartile range increase in PM_2.5_ and O_3_ below the NAAQS^¶^.

| ECG parameter | Lag (day) | PM_2.5_ | O_3_ |
| --- | --- | --- | --- |
| PR | 0 | -0.10 (-0.29, 0.08) | -0.01 (-0.29, 0.26) |
|  | 1 | -0.08 (-0.27, 0.10) | 0.06 (-0.22, 0.34) |
|  | 2 | -0.02 (-0.20, 0.17) | -0.02 (-0.29, 0.26) |
|  | 3 | 0.18 (0.00, 0.37) ^*^ | 0.09 (-0.18, 0.37) |
|  | 4 | 0.08 (-0.10, 0.27) | 0.29 (0.01, 0.56) ^*^ |
|  | 04 | 0.03 (-0.26, 0.32) | 0.18 (-0.23, 0.58) |
|  |  |  |  |
| QRS | 0 | 0.11 (-0.02, 0.24) | -0.10 (-0.29, 0.09) |
|  | 1 | 0.02 (-0.11, 0.15) | -0.05 (-0.25, 0.14) |
|  | 2 | -0.02 (-0.15, 0.11) | -0.09 (-0.28, 0.11) |
|  | 3 | 0.02 (-0.11, 0.15) | -0.02 (-0.21, 0.18) |
|  | 4 | 0.09 (-0.03, 0.22) | 0.13 (-0.06, 0.33) |
|  | 04 | 0.11 (-0.09, 0.31) | -0.05 (-0.34, 0.23) |
|  |  |  |  |
| QTc | 0 | 0.12 (0.02, 0.22) ^*^ | 0.19 (0.04, 0.34) ^*^ |
|  | 1 | 0.06 (-0.04, 0.16) | 0.22 (0.07, 0.38) ^**^ |
|  | 2 | 0.03 (-0.07, 0.13) | 0.08 (-0.07, 0.23) |
|  | 3 | 0.07 (-0.03, 0.17) | -0.02 (-0.17, 0.13) |
|  | 4 | 0.11 (0.01, 0.21) ^*^ | -0.01 (-0.16, 0.14) |
|  | 04 | 0.19 (0.03, 0.35) ^*^ | 0.20 (-0.02, 0.42) |
|  |  |  |  |
| HR | 0 | 0.15 (-0.16, 0.46) | 0.00 (-0.46, 0.46) |
|  | 1 | 0.41 (0.10, 0.73) ^**^ | 0.28 (-0.19, 0.76) |
|  | 2 | 0.16 (-0.15, 0.47) | 0.12 (-0.35, 0.60) |
|  | 3 | -0.19 (-0.50, 0.11) | -0.36 (-0.82, 0.11) |
|  | 4 | 0.08 (-0.23, 0.39) | -0.13 (-0.59, 0.34) |
|  | 04 | 0.29 (-0.19, 0.78) | -0.04 (-0.72, 0.65) |

CI: confidence interval; ECG: electrocardiogram; PM_2.5_: particulate matter ≤ 2.5 µm in aerodynamic diameter; O_3_: ozone; NAAQS: U.S. National Ambient Air Quality Standards; QTc: heart rate-corrected QT interval; HR: heart rate.

^¶^ Number of ECGs with exposure below the NAAQS (35 µg/m^3^ for PM_2.5_ and 70 ppb for O_3_) were 26255 on 5205 participants.

^*^ p-Value <0.05; ^**^ p-Value <0.01.

Participants without ECG measurement during the study period (2001-2012)

N = 907

Participants having ECG with the diagnosis of atrial fibrillation, atrial flutter, multifocal atrial tachycardia, or paced rhythms, or with non-physiological parameter values

N = 344

Participants without complete data on investigated ECG parameters or covariates in the main model, or whose home addresses were not available for geocoding

N = 44

N = 344

Participants with bundle branch block

N = 489

CATHGEN Cohort

Participants residing in NC N = 7116

N = 7116

Participants for analysis

N=5332

### **Figure S1.** Flow chart of the exclusion procedure.

NC: North Carolina; ECG: Electrocardiogram.


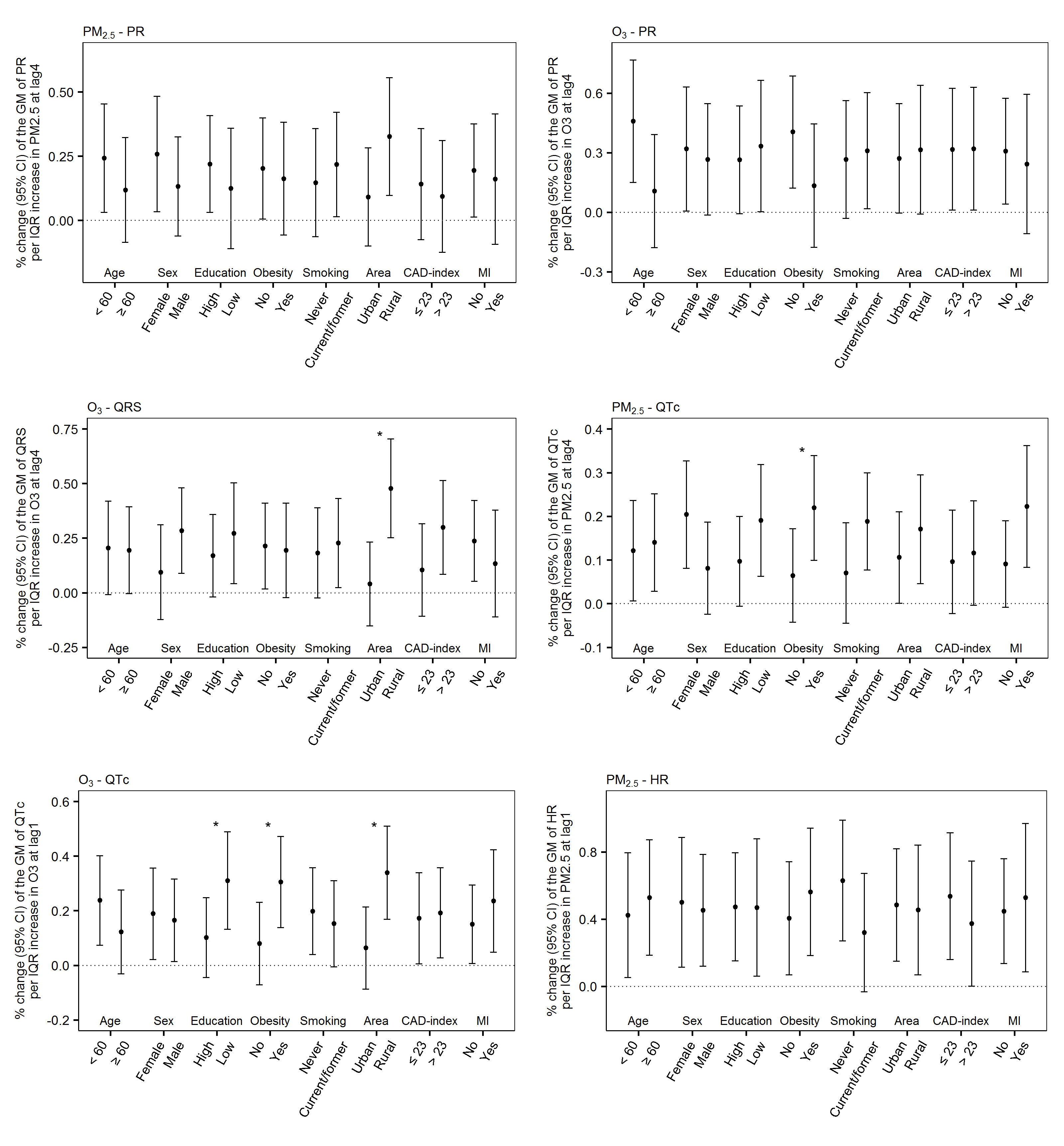
 **Figure S2.** Effect modification (percent change with 95% CI) by participant characteristics on the associations of air pollution with ECG parameters.

^*^ p-Value <0.05.

CI: confidence interval; ECG: Electrocardiogram; PM_2.5_: particulate matter ≤ 2.5 µm in aerodynamic diameter; O_3_: ozone; GM: geometric mean; IQR: interquartile range; QTc: heart rate-corrected QT interval; HR: heart rate; CAD: coronary artery disease.


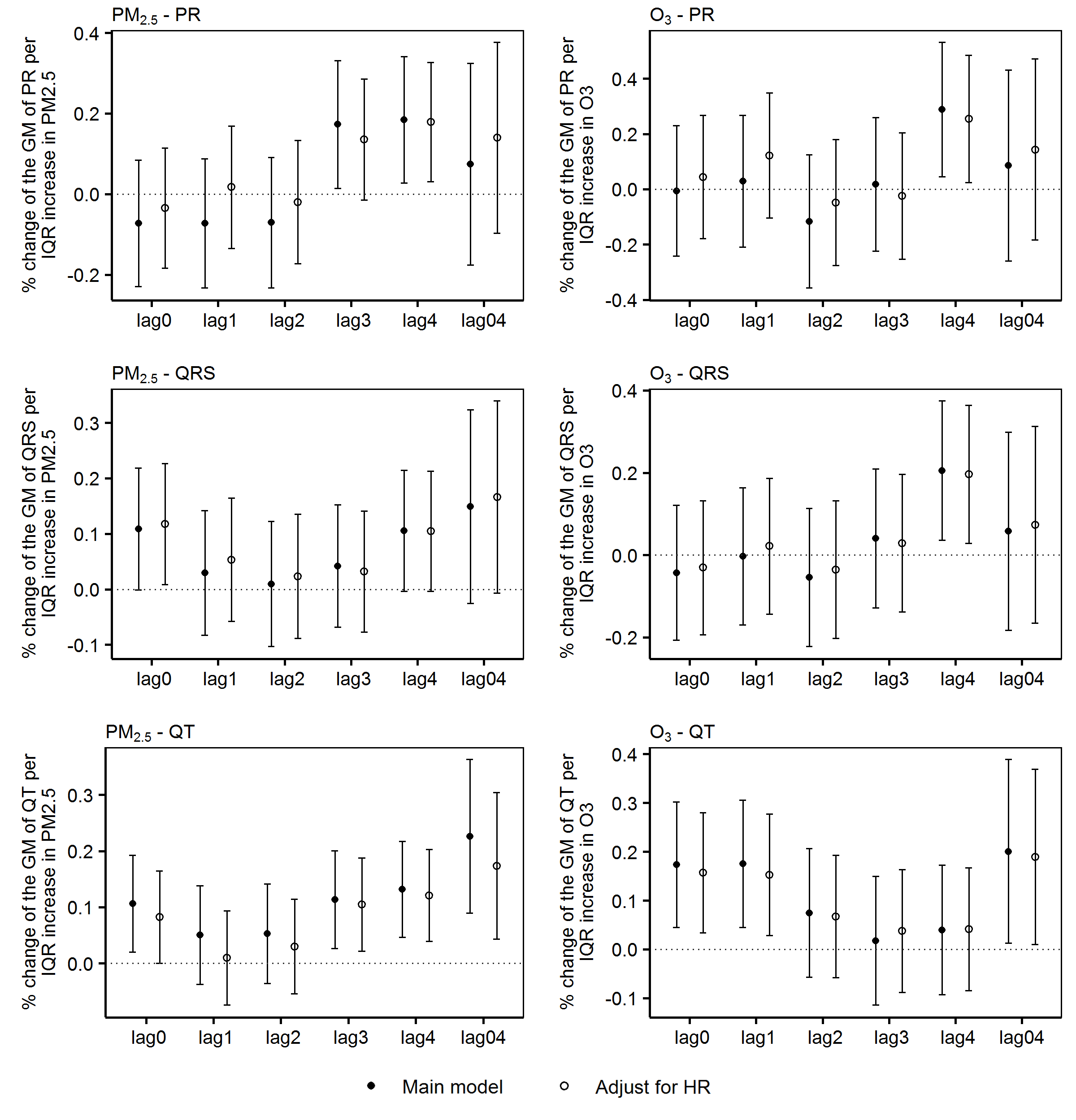


**Figure S3.** Percent change (95% CI) of the geometric mean of the PR, QRS, and raw QT intervals per interquartile range increase in PM_2.5_ and O_3_ in models with adjustment for HR.

CI: confidence interval; PM_2.5_: particulate matter ≤ 2.5 µm in aerodynamic diameter; O_3_: ozone; HR: heart rate; GM: geometric mean; IQR: interquartile range.


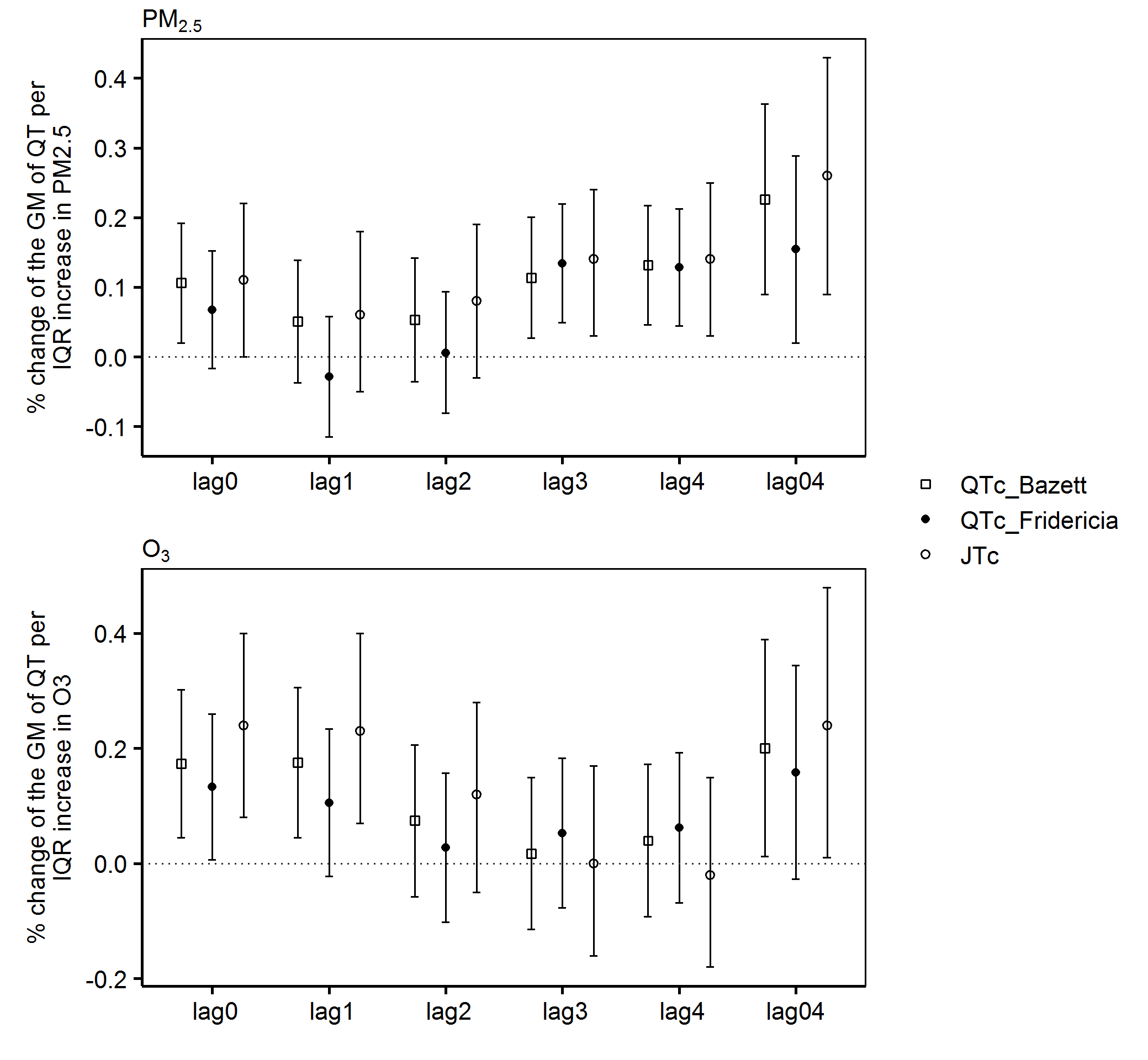


**Figure S4**. Comparison of the air pollution effects (percent change with 95% CI) on different ventricular repolarization indicators.

CI: confidence interval; PM_2.5_: particulate matter ≤ 2.5 µm in aerodynamic diameter; O_3_: ozone; GM: geometric mean; IQR: interquartile range; QTc_Bazett: heart rate-corrected QT interval calculated using the Bazett formula; QTc_Fridericia: heart rate-corrected QT interval calculated using the Fridericia formula; JTc: corrected JT interval calculated by subtracting QRS from QTc_Bazett.


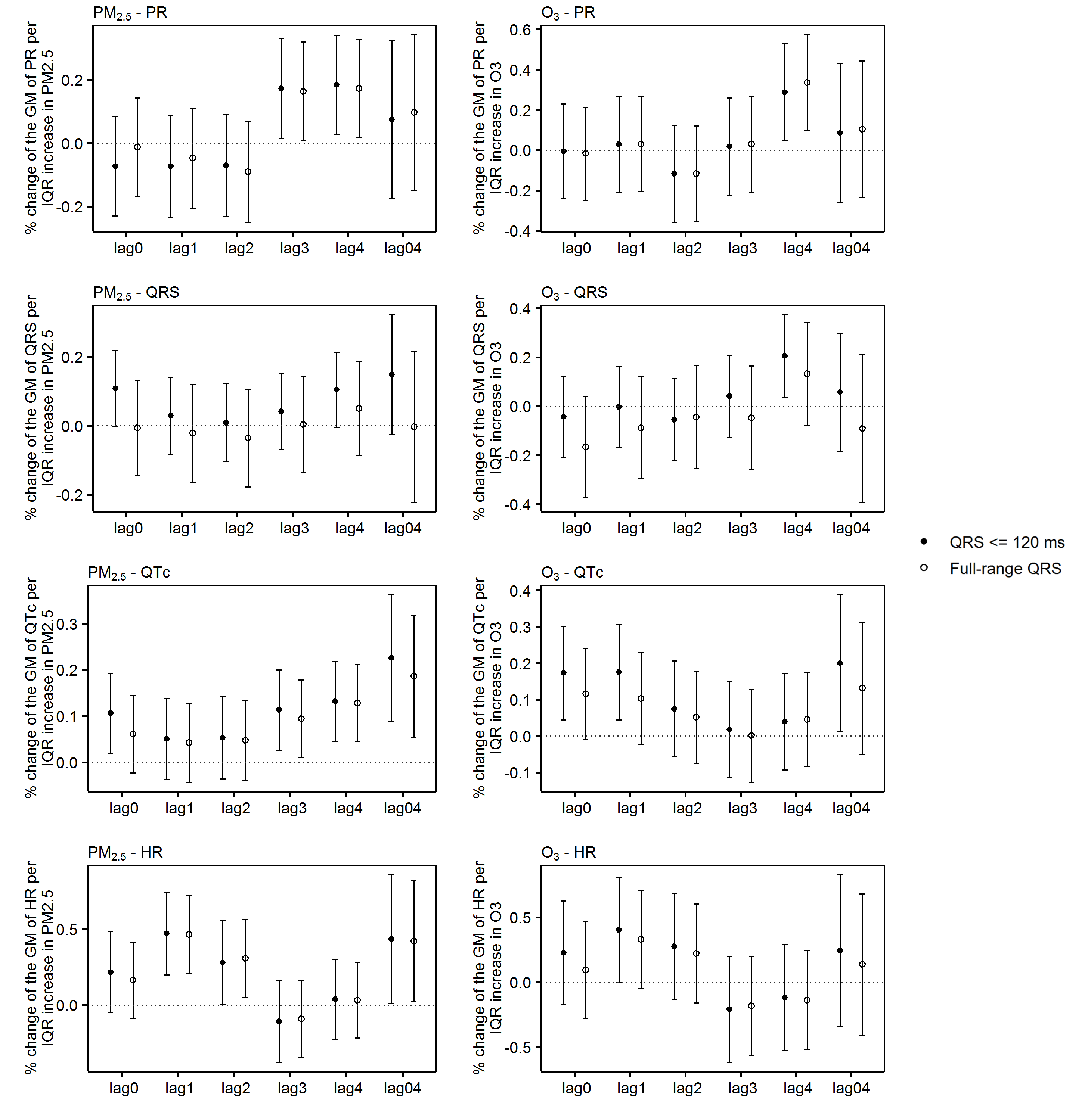


**Figure S5.** Percent change (95% CI) of the geometric mean of ECG parameters per interquartile range increase in PM_2.5_ and O_3_ among participants with QRS ≤ 120 ms and participants with QRS in the full range (50 ms ≤ QRS ≤ 170 ms).

CI: confidence interval; ECG: Electrocardiogram; PM_2.5_: particulate matter ≤ 2.5 µm in aerodynamic diameter; O_3_: ozone; HR: heart rate; GM: geometric mean; IQR: interquartile range.


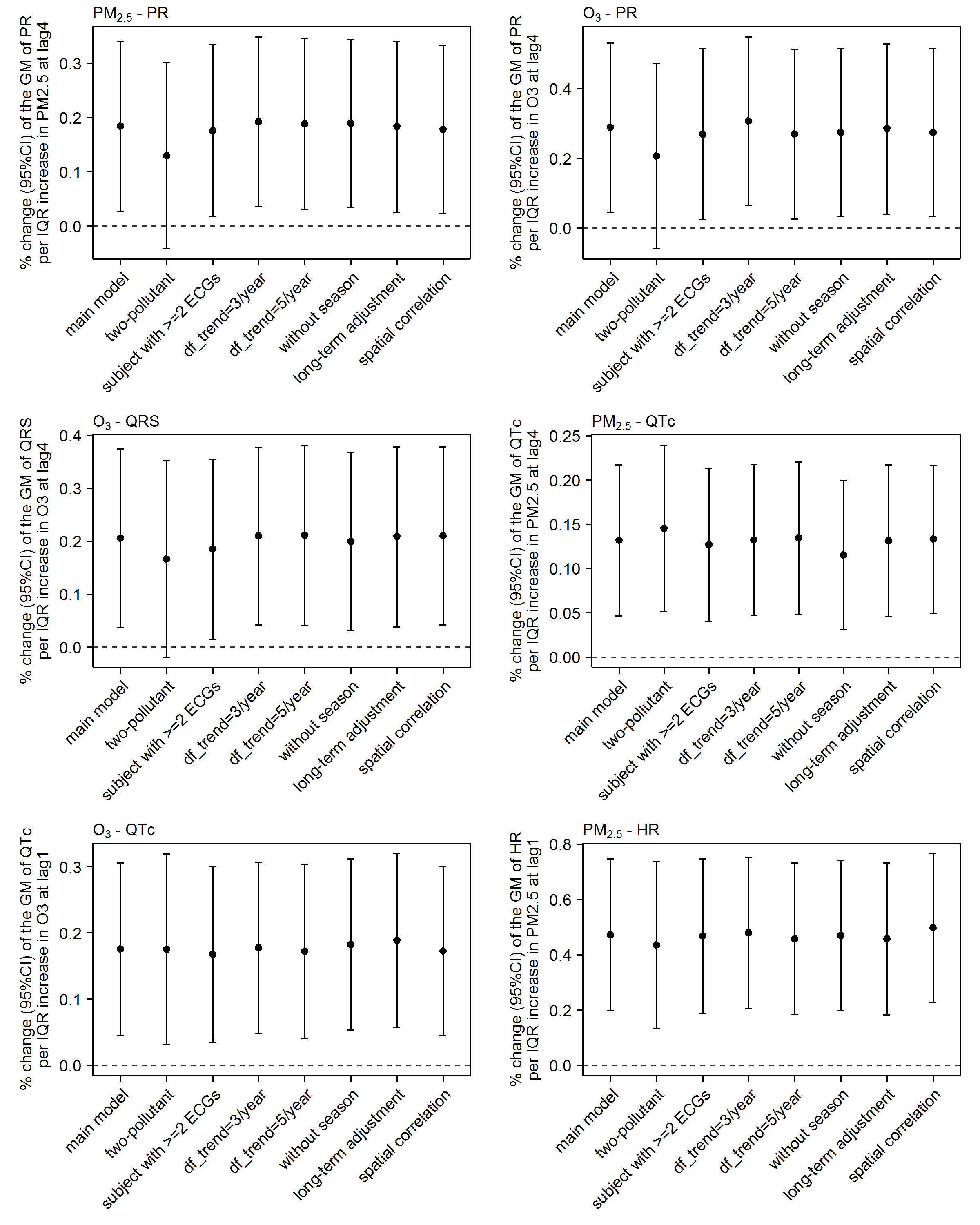


### **Figure S6.** Percent change (95% CI) of the geometric mean of ECG parameters per interquartile range increase in PM_2.5_ and O_3_ in sensitivity analyses.

CI: confidence interval; ECG: Electrocardiogram; PM_2.5_: particulate matter ≤ 2.5 µm in aerodynamic diameter; O_3_: ozone; QTc: heart rate-corrected QT interval; HR: heart rate; GM: geometric mean; IQR: interquartile range.

The order of effect estimates in each panel: (1) Main single-pollutant model; (2) two-pollutant model; (3) restricted to participants with two or more ECG measurements; (4) degree of freedom of time trend = 3/year; (5) degree of freedom of time trend = 5/year; (6) regression models without adjustment for season; (7) regression model with adjustment for long-term exposure; (8) regression model using spatial correlation structure of ECGs.
